# Autoimmune pulmonary alveolar proteinosis exacerbated by steroid therapy due to misdiagnosis as anti-aminoacyl-tRNA synthetase (ARS) antibody positive- interstitial pneumonia: a case report

**DOI:** 10.1186/s12890-022-01909-z

**Published:** 2022-03-31

**Authors:** Hiroshi Ishimoto, Noriho Sakamoto, Hirokazu Yura, Atsuko Hara, Takashi Kido, Hiroyuki Yamaguchi, Kazuko Yamamoto, Yasushi Obase, Yuji Ishimatsu, Minoru Satoh, Hiroshi Mukae

**Affiliations:** 1 Department of Respiratory Medicine, Nagasaki University Graduate School of Biomedical Sciences, 1-7-1 Sakamoto, Nagasaki, 852-8501 Japan; 2 Department of Respiratory Medicine, National Hospital Organization Nagasaki Medical Center, 2-1001-1 Kubara, Ohmura, Nagasaki 856-8562 Japan; 3 Department of Nursing, Nagasaki University Graduate School of Biomedical Sciences, 1-7-1 Sakamoto, Nagasaki, 852-8520 Japan; 4 Department of Clinical Nursing, School of Health Sciences, University of Occupational and Environmental Health, 1-1 Iseigaoka, Yahatanishi-ku, Kitakyushu, Fukuoka 807-0804 Japan

**Keywords:** Anti-ARS antibody, Anti-PL-7 antibody, Autoimmune alveolar proteinosis, Steroid, Case report

## Abstract

**Background:**

Anti-aminoacyl-tRNA synthetase (anti-ARS) antibodies are myositis-specific autoantibodies that have been identified in a subset of patients with interstitial pneumonia who do not present with dermatomyositis or polymyositis. Anti-ARS antibody-positive interstitial pneumonia is commonly treated with steroids or immunosuppressive agents and is usually responsive to these therapies. Here, we present in detail a case in which respiratory failure of a patient diagnosed with anti-ARS antibody-positive interstitial pneumonia was exacerbated by treatment with steroids and immunosuppressive agents. Further examination revealed misdiagnosis of this patient and a subsequent diagnosis of autoimmune pulmonary alveolar proteinosis.

**Case presentation:**

A 66-year-old man presented to the hospital with dyspnea on exertion, which resulted in the detection of interstitial pneumonia. Serum anti-ARS antibodies were detected; however, there were no other findings suggestive of myositis. Pulmonary alveolar proteinosis (PAP) was suspected based on the marked increase in serum KL-6 and chest computed tomography findings. The bronchoalveolar lavage revealed no milky changes in the lavage fluid. After treatment with steroids and initiation of immunosuppressive agents for anti-ARS antibody-positive interstitial pneumonia, respiratory failure and chest imaging findings showed worsening of the condition. Bronchoscopy was repeated, and milk-like alveolar lavage fluid was collected; serum anti-granulocyte macrophage colony-stimulating factor antibody was identified. Steroids and immunosuppressive agents were gradually tapered and discontinued, and the patient’s condition stabilized after repeated alveolar lavage under general anesthesia.

**Conclusion:**

Due to similar presentation, PAP can be misdiagnosed as interstitial pneumonia. If pulmonary lesions due to interstitial pneumonia are exacerbated by immunosuppressive treatment, physicians should reconsider the diagnosis and include PAP in the differential diagnosis.

## Background

Pulmonary alveolar proteinosis (PAP) is attributed to the accumulation of surfactant-derived lipoprotein compounds in the alveolar space owing to disturbed macrophage differentiation and function [1]. Approximately 90% of cases are autoimmune PAP (APAP), which is diagnosed by the detection of anti-granulocyte macrophage-colony stimulating factor (GM-CSF) antibodies in the serum [2, 3]. Anti-GM-CSF antibodies play a pivotal role in the disturbance of macrophage differentiation and function in the lungs of patients with APAP. The standard therapy is whole lung lavage [3], with inhaled GM-CSF therapy having potential as a future treatment [4].

Anti-aminoacyl-tRNA synthetase (anti-ARS) antibodies are myositis-specific autoantibodies that have been identified in a subset of patients with interstitial pneumonia who do not present with dermatomyositis or polymyositis [5, 6]. Interstitial pneumonia with anti-ARS antibody is commonly treated with steroids or immunosuppressive agents and is usually responsive to these therapies [7].

In this report, we describe the case of a patient with APAP who had been treated with steroids and immunosuppressive agents for interstitial pneumonia associated with anti-ARS antibody positivity.

## Case presentation

**Fig. 1:**
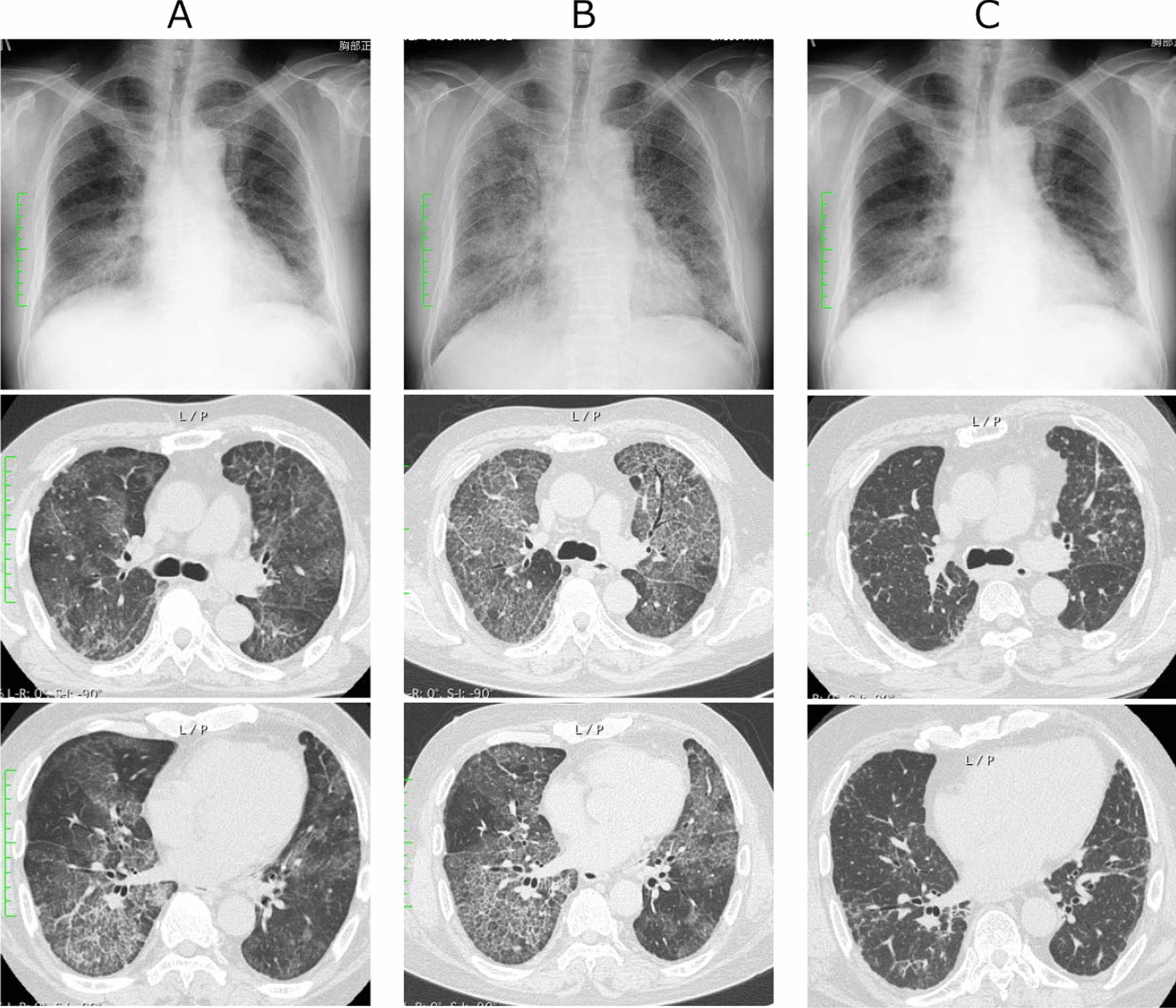
Chest radiography and computed tomography findings. First visit to the previous hospital, 2 months before referral to our hospital (**A**). At the time of referral to our hospital, the reticular shadows were enlarged on chest radiography and the crazy paving appearance was enlarged and well-defined on chest computed tomography (**B**). One year later, the chest imaging findings have improved following the reduction and discontinuation of steroids and immunosuppressive agent and repeated total lung lavage under general anesthesia (**C**)

A 66-year-old Japanese man presented with dyspnea on exertion for the previous 4 months and had visited a different hospital 2 months prior to his presentation at our hospital. His smoking history was 0.5 pack year. Chest radiography revealed small nodular and reticular shadows, predominantly in the lower lung fields, and a chest computed tomography scan revealed diffuse ground-glass shadows and partial interlobular septal thickening, known as crazy paving appearance (Fig. 1A). Serum KL-6 was markedly elevated at 10,513 U/mL; however, the bronchoalveolar lavage fluid (BALF) was not milky in appearance, and the BALF cell analysis revealed elevated lymphocytes (82%). In addition, anti-ARS antibody in the serum was detected; thus, a diagnosis of interstitial pneumonia with positive anti-ARS antibodies was made in the patient; prednisolone and cyclosporine treatment were initiated. Following treatment initiation, the patient rapidly developed respiratory failure, and he was transferred to our hospital 1 month after prednisolone and cyclosporine were initiated. Vital signs on admission were: body temperature, 37.1 °C; pulse, 88 beats per minute; blood pressure, 130/93 mmHg; and oxygen saturation (SpO_2_), 93% with 15 L/min O_2_ inhalation using a reservoir mask. On chest auscultation, fine crackles were heard in both lungs. There was no muscle grasp pain or weakness, and no skin findings suggestive of dermatomyositis, such as heliotrope rash or Gottron’s sign, were noted. An arterial blood gas analysis (measured when the patient was on 15 L/min of oxygen through a reservoir mask) showed a pH of 7.460, PaCO_2_ of 36.1 mmHg, PaO_2_ of 69.2 mmHg, and HCO_3_ of 25.4 mmol/L. Laboratory findings are shown in Table 1. KL-6 and carcinoembryonic antigen levels were > 5000 U/mL and 19.4 ng/mL, respectively. A commercial anti-ARS antibody assay (mixture of Jo-1, PL-7, PL-12, EJ, and KS antigens) was positive, but tests for anti-nuclear antibodies and other specific autoantibodies, including anti-Jo-1, were negative. Subsequently, the anti-ARS antibody was evaluated by immunoprecipitation and found to be an anti-PL-7 antibody. Chest radiography revealed an enlargement of diffuse reticular shadows, and chest computed tomography revealed a wide distribution of well-defined crazy-paving appearance (Fig. 1B). We suspected PAP and decided to perform a bronchoscopy. The patient’s respiratory condition was poor; bronchoscopy was performed under ventilator control with tracheal intubation. Bronchoalveolar lavage revealed cloudy milk-like fluid. Serum anti-GM-CSF antibody level was 5.2 µg/mL (< 1.0 µg/mL), and APAP was diagnosed. Thereafter, prednisolone and cyclosporine were tapered off. Total lung lavage under general anesthesia was performed twice for both lungs over a period of 6 months, resulting in resolution of respiratory failure. An arterial blood gas analysis (measured when the patient was on 1 L/min of oxygen through a nasal canula) showed a pH of 7.400, PaCO_2_ of 43.5 mmHg, PaO_2_ of 86.7 mmHg, and HCO_3_ of 26.4 mmol/L) and findings in radiographic images improved (Fig. 1C).

**Table 1:** Laboratory findings on admission

Laboratory findings		Reference value
WBC (×10^3^/μL)	15.2	3.3–8.6
RBC (×10^6^μL)	6.42	4.35–5.55
Hemoglobin (g/dL)	18.5	13.7–16.8
Hematocrit (%)	53.8	40.7–50.1
Platelet (μL)	174	158–348
TP (g/dL)	6.4	6.6–8.1
Albumin (g/dL)	3.3	4.1–5.1
AST (IU/L)	37	13–30
ALT (IU/L)	29	10–42
LDH (IU/L)	385	124–222
BUN (mg/dL)	21	8–20
Creatinine (mg/dL)	0.71	0.65–1.07
Creatine kinase (U/L)	21	59–248
Aldolase (U/L)	10.1	2.7–7.5
CRP (mg/dL)	0.07	0.00–0.14
KL-6 (U/mL)	> 5000	105.3–401.2
SP-A (ng/mL)	203.9	< 43.8
SP-D (ng/mL)	432	< 110
CEA (ng/mL)	19.4	< 5.0
Anti-nuclear antibodies (n times)	< 80	< 80
Anti-ARS antibody (INDEX)	157	< 25
Anti-CCP antibody (U/mL)	< 0.6	< 4.5
Anti-SS-A antibody (U/mL)	1.0	< 10
Anti-SS-B antibody (U/mL)	< 1.0	< 10
MPO-ANCA (U/mL)	< 1.0	< 3.5
PR3-ANCA (U/mL)	< 1.0	< 3.5

## Discussion and Conclusions

The patient in the present case had both anti-GM-CSF and anti-ARS antibodies. The incidence of autoimmune diseases in patients with PAP is low, reportedly 1.4–1.7% of all PAP cases [2, 8]. Therefore, the coexistence of anti-GM-CSF and anti-ARS antibodies in the present case could be coincidental. In contrast, the prevalence of anti-GM-CSF antibodies in patients with autoimmune diseases including polymyositis is reportedly 9.6% [9]. In addition, a study of patients with anti-ARS antibody-positive interstitial pneumonia demonstrated that patients with progressive interstitial pneumonia had higher serum GM-CSF levels than those with stable interstitial pneumonia [10]. The exact mechanism of the production of anti-GM-CSF antibody in patients with APAP is unknown; however, it could be speculated that a chronic condition associated with production of anti-ARS antibodies could have resulted in an increase in GM-CSF, which in turn triggered the production of anti-GM-CSF antibodies.

The pathophysiology of the coexistence of autoimmune diseases and PAP is not clear; however, the comorbidity of autoimmune diseases and PAP can cause serious therapeutic problems. APAP, which accounts for 90% of the PAP cases, is caused by the production of anti-GM-CSF antibodies, and inhaled GM-CSF therapy is expected to be beneficial for these patients [4]. Anti-ARS antibodies, known to be associated with dermatomyositis and polymyositis, are also detected in 6.0–7.6% of patients with idiopathic interstitial pneumonia [5, 6]. Among the anti-ARS antibodies detected in patients with idiopathic interstitial pneumonia, anti-PL-7 antibody is the second most common, after anti-Jo-1 [11]. Anti-ARS antibody-positive interstitial pneumonia is known to respond well to treatment with steroid and immunosuppressive agents [12]. Conversely, a cohort study on APAP, wherein most steroid-using cases were initially diagnosed with interstitial lung disease and treated with steroids, demonstrated that steroid therapy exacerbated respiratory failure [13]. In some cases, PAP exacerbated during steroid treatment of interstitial pneumonia associated with dermatomyositis or polymyositis [14, 15].

In conclusion, PAP can be misdiagnosed as interstitial pneumonia due to similar presentation. If pulmonary lesions due to interstitial pneumonia are exacerbated by immunosuppressive treatment, physicians should reconsider the diagnosis and include PAP in the differential diagnosis.

## Data Availability

Not applicable.

## Abbreviations

APAP: Autoimmune pulmonary alveolar proteinosis
ARS: Aminoacyl-tRNA synthetase
BALF: Bronchoalveolar lavage fluid
GM-CSF: Granulocyte macrophage-colony stimulating factor
PAP: Pulmonary alveolar proteinosis
